# The effectiveness of social media-based microlearning in improving knowledge, self-efficacy, and self-care behaviors among adult patients with type 2 diabetes: an educational intervention

**DOI:** 10.1186/s12902-024-01626-0

**Published:** 2024-06-28

**Authors:** Samira Rahbar, Nahid Zarifsanaiey, Manoosh Mehrabi

**Affiliations:** 1 E-learning in Medical Sciences, Shiraz University of Medical Sciences, Shiraz, Iran; 2 Department of E-learning, Virtual School, Comprehensive Centre of Excellence for E-Learning in Medical Sciences, Shiraz University of Medical Sciences, Shiraz, Iran

**Keywords:** Knowledge, Self-care, Self-efficacy, Microlearning, Type 2 diabetes, Diabetes clinic, Social media

## Abstract

**Background:**

The purpose of this study is to investigate the impact of social media-based microlearning (SMBM) on enhancing the knowledge, self-care, and self-efficacy behaviors of patients with type 2 diabetes (T2D) receiving care at a hospital-based diabetes clinic in Zahedan, Iran.

**Methods:**

This intervention study was conducted from September 2021 to the end of 2022, with an intervention group (SMBM) and a control group (conventional-based training) consisting of patients with T2D. A total of 80 eligible patients were selected using a convenience sampling method and randomly assigned to either the intervention group (*n* = 40) or the control group (*n* = 40). The knowledge level, self-care, and self-efficacy of the samples were assessed before and two weeks after the educational intervention. Data analysis was conducted using SPSS version 24, and independent and paired T-tests were used for analysis.

**Results:**

The results of the study revealed that after the intervention, the levels of knowledge, self-care, and self-efficacy in the intervention group were significantly higher than those in the control group (p-value < 0.001).

**Conclusion:**

In conclusion, the SMBM appears to be an effective tool for improving self-efficacy, self-care, and knowledge among patients with type 2 diabetes.

## Background

Type 2 diabetes (T2D) is a rapidly growing global health concern, demanding effective management strategies that empower patients [1, 2]. Effective diabetes management relies on two key factors: self-care and self-efficacy. Self-care encompasses daily actions like blood sugar monitoring, healthy eating, and exercise, while self-efficacy is a patient’s confidence in managing their condition. Self-efficacy is defined as an individual’s belief in their ability to perform an action successfully, and it plays a role in the clinical outcome of diabetes management [3–6]. Both self-care and self-efficacy play integral roles in diabetes management, underscoring the importance of addressing them in comprehensive diabetes interventions [7–10]. Over 98% of diabetes care is provided by patients and their families, making it essential for patients to have sufficient knowledge and skills in this area. To effectively treat diabetes, patients must have adequate knowledge and skills, and diabetic education aims to improve individual self-efficacy and management ability over their situation [11, 12]. Given the significance of these components mentioned, it can be inferred that patient education is a vital aspect of diabetes care and plays a crucial role in disease management [13, 14]. Different educational methods have varying effects on different groups and individuals, and it is essential to measure these effects. Traditional in-person education methods, while valuable, can be time-consuming and expensive to deliver, limiting their reach [15].

Microlearning emerges as a powerful alternative to traditional patient education. By breaking down complex medical information into digestible modules, microlearning empowers patients to learn and retain information incrementally, fostering a sense of control over their diabetes management [16, 17]. Engaging formats like short videos, infographics, and audio clips make learning more captivating. Social media platforms take microlearning to the next level [18]. This synergy between microlearning and social media offers unique advantages for diabetes education, creating a dynamic learning environment known as Social Media-Based Microlearning (SMBM) [19].

SMBM empowers patients in several key ways. Firstly, it allows them to access educational content directly relevant to their specific needs [20]. Secondly, SMBM fosters a personalized learning experience by allowing patients to progress at their own pace. These features are further enhanced by the interactive nature of social media platforms [21]. Through these platforms, patients can connect with healthcare professionals and peers facing similar challenges, fostering a supportive online community [15]. Finally, SMBM’s content is readily available on mobile devices, allowing patients the flexibility to learn on the go, whenever it suits their schedule [22]. Research confirms the effectiveness of this approach, with studies demonstrating SMBM’s success in boosting knowledge, self-care behaviors, and self-efficacy in managing diabetes across various populations [23, 24].

While research suggests promise for SMBM, some studies have compared it to in-person education and found in-person approaches to be more effective. Studies have shown that in-person diabetes education programs can lead to significant improvements self-care behaviors, compared to control groups receiving no education [25].

Despite these challenges, the potential benefits of SMBM make it a valuable tool for diabetes education. By carefully managing social media platforms for learning, healthcare providers can empower patients with the knowledge and confidence to effectively manage their T2D [26].While previous research has explored the general benefits of SMBM for diabetes education, it fails to capture the specific needs and context of this local population. This lack of understanding is particularly concerning because cultural and socioeconomic factors can significantly influence the way patients receive and engage with health information. Without local data on the effectiveness of SMBM, healthcare providers in Zahedan may struggle to develop targeted and culturally appropriate educational strategies. This could lead to suboptimal diabetes management outcomes for patients, potentially impacting their health and well-being. This study aims to address this critical gap in knowledge by investigating the impact of SMBM on knowledge, self-efficacy, and self-care behaviors among adult patients with T2D in Zahedan.

## Method

### Study design

This study is a pretest-posttest interventional study was conducted on one intervention group (SMBM) and a control group (conventional method).

#### Participants

### Eligibility criteria for participants

This study will recruit participants from the Zahedan Diabetes Clinic following strict inclusion and exclusion criteria. To be included, patients must be 18 years or older with a confirmed diagnosis of Type 2 Diabetes (T2D) by a physician for at least one year. Additionally, they should have a documented history of visiting the clinic, demonstrate adequate visual and auditory capabilities, and express willingness to participate throughout the study. Notably, data analysis will exclude participants who are unwilling to cooperate at any stage of the study or who miss two or more social media training sessions.

### Setting

The study population consisted of adults with T2D who attended diabetes clinics affiliated with Zahedan University of Medical Sciences from October 2021 to December 2022.

### Intervention group

Following informed consent and pre-test completion, participants were randomly assigned to either the intervention or control group. Both groups received usual care and training, including a three-hour workshop on healthy eating, medication adherence, and blood sugar monitoring.

The intervention group received additional support through a two-week intervention delivered via a private WhatsApp group. This involved creating and delivering bite-sized, digestible educational content specifically designed for the platform. Participants received a mix of engaging microlearning materials uploaded daily between 6 PM and 8 PM for two weeks (Table 1). These materials included:

- Ten 4-minute audio podcasts: Condensed and informative lessons on key diabetes management topics.
- Twenty-five short text messages: Quick reminders, tips, and educational points.
- Twelve text messages with photos: Combining visuals with text for enhanced engagement.
- Four 5-minute video messages: Short clips demonstrating or explaining specific concepts.

Beyond content delivery, the WhatsApp group fostered a sense of community and interactivity. Researchers facilitated discussions at designated times, addressing questions and concerns. Participants were encouraged to share experiences, ask questions of peers, and offer support. This interactive environment promoted collaborative learning and knowledge sharing, a key strength of social media-based microlearning.

A professor with expertise in endocrinology, e-learning, nutrition, and fitness (10–15 years of experience) developed and facilitated these discussions, ensuring the quality and effectiveness of the intervention.

**Table 1:** The educational content provided was as follows

Educational Content	Topics Covered
Audio podcast, tutorial video, group discussion, photos with text messages	Initial definition of diabetes, diabetic needs, importance of self-care behaviors, acute and chronic side effects recognition
Audio podcast, tutorial video, group discussion, send photos with text, send messages	Nutrition guidance: soluble fibers, fruits, whole grains, meal frequency increase, salad and low-calorie food usage, fruit juice avoidance, sugar-free drinks and desserts, whole wheat flour substitution, fish oil and niacin avoidance, fat intake reduction, daily water intake recommendation
Audio podcast, tutorial video, group discussion, send photos with text, send messages	Diabetic foot care instructions: specialist visits, daily foot checks, protective shoe usage, foot hygiene practices like washing with lukewarm water and soap, drying feet properly, toenail trimming, appropriate socks and shoes selection
Audio podcast, tutorial video, group discussion, send photos with text, send messages	Exercise guidelines: aerobic and anaerobic exercises explanation, optimal exercise timing and duration, exercise precautions and post-exercise care
Group discussion by researchers and patients’ own company	An overview of the presented material

### Control group

There was no intervention for the control group, and the group had the same routine care as the diabetes center. Routine in-person education includes a three-hour workshop that dives deep into diabetes education. The workshop covers various topics such as the description of the disease, its symptoms, and potential complications. Additionally, it delves into treatment approaches, including the different types of insulin and proper storage techniques.

Two week after finishing the training, participants from both groups took the posttest. By the study’s conclusion, educational material was provided to the control group.

### Sample size & randomization

Based on a statistical population of 1600 patients referred to the diabetes clinic of ZUMS, and assuming a confidence rate of 95%, an estimated error rate of 5/0.05, and a z-value of 96/1.96, 1600 participants were chosen for the study. Given the results of similar studies conducted by Nasser et al. (2023) and the formula for comparing the means of two groups, a sample size of 80 (40 in the intervention group, 40 in the control group) was determined to be sufficiently representative.

$$n=\frac{{\left({Z}_{1-\frac{\alpha }{2}}+{Z}_{1-\beta }\right)}^{2}({\delta }_{1}^{2}+{\delta }_{2}^{2})}{{({\mu }_{1}-{\mu }_{2})}^{2}}$$

Eighty patients were selected based on the convenient sampling method. In order to achieve the volume of the sample, the researcher referred to the clinic on weekdays and, while describing the goals of the work, invited patients with study entry requirements to participate in the study. Using a simple random table, 40 patients were randomly assigned to each group in either school.

## Data collection tools

### Demographic information questionnaire

This questionnaire included demographic profile (age, gender, marital status, education level, occupation, household income, duration of illness).

### Michigan diabetic knowledge questionnaire (MDKQ-1)

The MDKQ-1 is a comprehensive tool designed to evaluate patients’ understanding of diabetes, encompassing key areas such as diet, blood sugar management, physical activity, medication usage, and potential complications. This questionnaire comprises 20 questions and employs a scoring mechanism based on a Likert scale (3 points for correct responses, 2 points for “I don’t know,” and 1 point for incorrect answers). The total score is derived by summing the individual question scores, ranging from 0 to 40 points. Higher scores reflect a deeper knowledge level, while lower scores indicate a need for further education. Developed by Rahimian et al. in 2011, the MDKQ-1 was specifically crafted to explore the direct and indirect influences of diabetic knowledge and social support on self-management among 500 T2D outpatients in Tehran. The questionnaire’s content validity was established through expert evaluation, and its reliability was assessed using Cronbach’s Alpha, yielding an impressive coefficient of 0.93 for the entire questionnaire [26].

### Diabetes self-care questionnaire 2 (DSCQ-2)

The Diabetes Self-Care Questionnaire 2 (DSCQ-2) was developed by Souza et al. in 2009 to assess the self-care practices of diabetic patients. This questionnaire comprises 14 questions, including 9 related to weekly self-management, 2 to monthly self-management, and 3 to annual self-management. The scoring system uses a Likert scale, with options ranging from 1 (I have no idea) to 4 (I completely agree). The total score is the sum of all the questions, resulting in a range between 35 and 140 points, where higher scores indicate better self-care practices.

The DSCQ-2 includes the following sections:

- Weekly Self-Care Scale: This scale consists of 9 components, including medical treatment and medication, general diet, exercise, self-contained sugar management, and leg care. Patients are asked to determine the extent of their self-care activities over the past week.
- Monthly Self-Care Scale: This scale includes 2 questions related to blood sugar control and self-management practices. Patients are asked to assess the percentage of their respective actions performed over the past 6 months.
- Annual Self-Care Scale: This scale consists of 3 questions related to long-term diabetes management, specifically eye, kidney, and heart complications.

The same questionnaire was also employed in a 2011 study by Bougar et al. This study investigated the direct and indirect influences of diabetic knowledge and social support on self-management behaviors among 500 outpatients with type 2 diabetes in Tehran. Bougar et al. (2011) established the questionnaire’s internal consistency using Cronbach’s alpha. The alpha coefficients indicated good reliability, with values of 0.95 for weekly self-care, 0.86 for monthly self-care, 0.67 for annual self-care, and 0.95 for the entire questionnaire [27].

### Diabetes self-efficacy scale

The Diabetes Self-Efficacy Scale consists of 10 items designed based on the Van Bijlert (1999) and Chen Zhu (2005) diabetes self-efficacy scales. This questionnaire was initially developed in a study by Rahimian et al. (2011) to investigate the direct and indirect effects of diabetes knowledge and social support on diabetes self-management in 500 individuals (245 men and 255 women) with T2D diabetes in Tehran. Some items were modified, resulting in a final questionnaire with 10 questions covering eight factors: treatment adherence (question 1), dietary plan (questions 2 and 3), exercise (questions 4 and 5), self-monitoring of blood sugar levels (question 6), foot care (question 7), blood sugar level management during hypoglycemia (question 8), prevention of blood sugar level fluctuations (question 9), and management of hyperglycemia (question 10). This scale ranges from never scoring zero to always scoring 100, with an internal consistency reported as Cronbach’s alpha of 0.96 [28].

### Statistical analysis

The data was analyzed by SPSS software (version 24).The normalization of the data was first measured using the Kolmogorov-Smirnov test. The mean, standard deviation, and range were analyzed for the indicators of abundance. For the independent T-test, the Kai test was used to compare the mean data in both groups (intervention and control) before and two weeks after the intervention. A P-value of 0.05 was considered statistically significant in all tests.

## Results

**Fig. 1:**
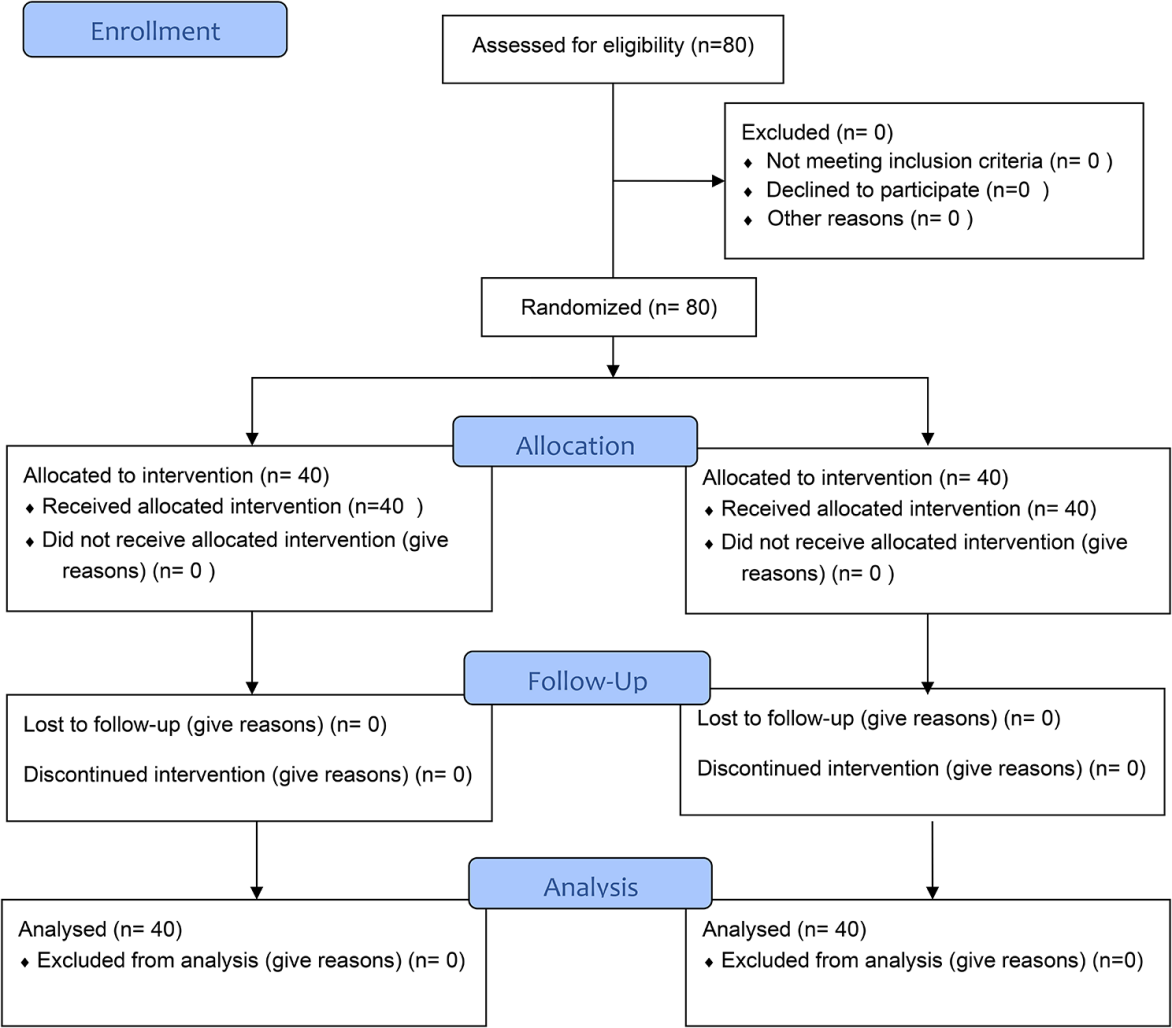
The patient’s recruitment flow diagram

In this study, a total of 80 patients were included in the research process, with 40 patients in the control group and 40 patients in the intervention group (Fig. 1).

To evaluate the difference between the two groups in terms of gender, marital status, education, occupation, marital status, and income, the Chi-square was used. The results revealed that there is no significant difference between the two groups in terms of gender, education, occupation, and income at a confidence level of 95%. To compare the age and duration of illness between the two groups, an independent T-test was used. The results indicated that there is no significant difference between the two groups in terms of age and duration of illness (Table 2).

**Table 2:** Participants’ demographic characteristics of patients among the intervention and control groups

Variable		Intervention	Control	*P*-value
age		Mean ± SD	Mean ± SD	0.73
		41.77 ± 5.66	40.25 ± 6.85	
Duration of Illness		Mean ± SD	Mean ± SD	0.09
		4.35 ± 1.45	3.77 ± 1.60	
Variable	Subgroup	N (%)	N (%)	P-value
Gender	Male	15 (37.5)	10 ( 25)	0.043
Marital status	Single	6 (15.3)	21 (51.2)	0.35
	Married	33 (82.5)	20 (48.7)	
Employment status	Occupation	12 (30)	13 (32.5)	0.750
	Housekeeper	10 (25)	10 (25)	
	Self-Employed	12(30)	11 (27.5)	
	unemployed	6( 15)	6( 15)	
Education level	Diploma	14 (35)	11(27.5)	0.84
	Advanced Diploma	12 ( 30)	16 ( 40)	
	Bachelor’s Degree	13 ( 32.5)	10 (25)	
	Postgraduate Degree	1 (2.5)	3 ( 7.5)	

The knowledge scores of the intervention and control groups were assessed and contrasted before and one week after the intervention (Table 3).

**Table 3:** Within- and between-group comparison of the knowledge Scores in Intervention and Control Groups Before and After the Educational Intervention

Intervention Group	Control group		Social media-based microlearning		Between-group comparison ((*p*-value)
	M	SD	M	SD	
Pre-test	36.12	8.54	35.95	10.95	0.93
Post- test	35.95	10.95	40.10	7.24	< 0.001
Within-group comparison (p-value)	0.98		< 0.001		---

The table shows that there was a statistically significant difference between the intervention and control groups on the post-test (p-value < 0.001). This means that the students in the intervention group scored significantly higher on the knowledge test after the intervention than the students in the control group. The table also shows that there was a statistically significant difference in the knowledge scores within the intervention group (p-value < 0.001). The results revealed that the knowledge score in the SMBM group was significantly higher than the control group two weeks after the intervention.

To determine the difference in the average level of the self-care score in the two intervention and control groups before and after the intervention, independent T-test was used (Table 4).

**Table 4:** Within- and between-group comparison of the self-care Scores in Intervention and Control Groups Before and After the Educational Intervention

Self-care	Social media-based microlearning *N*(40)				Control group *N* (40)			
	M		SD		M	SD		*P*-value
Pre-test	16		7.06		10/15	7.43		0.35
Post- test	22.42		5.75		15.52	7.05		< 0.001
**Within-group comparison**								
**Social media-based microlearning N(40)**				**P-value**	**Control group N(40)**			**P-value**
Pre-test		Post- test		< 0.001	Pre-test		Post- test	0.74
**Mean (SD)**		**Mean (SD)**			**Mean (SD)**		**Mean (SD)**	
16.6 (7.06)		22.42 (5.75)			15.1 (7.43)		15.52(7.05)	

As shown in the table above, the SMBM led to significant improvements in self-care behaviors compared to the control group, as indicated by the p-values (< 0.001). This suggests that utilizing social media for microlearning can positively impact self-care practices after two weeks, highlighting its effectiveness in promoting behavioral change.

To determine the difference in the average level of self-efficacy score in the two intervention and control groups before and after intervention, independent T-test was used (Table 5).

**Table 5:** Within- and between-group comparison of the self-efficacy Scores in Intervention and Control Groups Before and After the Educational Intervention

Dimensions	Social media-based microlearning *N*(40)			Control group *N*(40)		
	Mean	SD		Mean	SD	*P*-value
Pre-adherence to treatment	37.25	9.05		38.50	9.21	0.54
Post- adherence to treatment	67.75	8.91		35.00	8.77	< 0.001
Pre-dietary plan	74.50	17.53		69.00	12.96	0.11
Post- dietary plan	135.50	17.82		72.50	15.64	< 0.001
Pre-exercise	74.50	17.38		70.00	11.76	0.17
Post- exercise	134.98	17.82		63.00	12.44	< 0.001
Pre-self-monitoring of blood sugar levels	36.75	8.28		37.50	10.60	0.71
Post- self-monitoring of blood sugar levels	67.75	8.91		31.50	6.22	< 0.001
Pre-foot care	37.25	8.16		34.00	7.08	0.06
Post- foot care	67.75	8.91		31.46	5.22	< 0.001
Pre- blood sugar level management during hypoglycemia	38.25	8.76		36.00	5.90	0.15
Post- blood sugar level management during hypoglycemia	67.75	8.91		30.65	6.22	< 0.001
Pre- management of hyperglycemia	37.75	9.19		33.25	7.29	0. 18
Post- management of hyperglycemia	67.75	8.97		33.50	6.22	< 0.001
Pre-total self-efficacy	372.50	70.37		346.25	45.94	0.05
Post- total self-efficacy	677.50	89.11		328.00	61.10	< 0.001

Within comparison						
	Social media-based microlearning			Control group		
	Pre	Post		Pre	Post	
	Mean (SD)	Mean (SD)	P-value	Mean (SD)	Mean (SD)	P-value
Adherence to treatment	37.25 (9.05)	67.75 (8.91)	< 0.001	38.5 (9.21)	35 (8.77)	0.14
Dietary plan	74.5 (17.53)	135.5 (17.82)	< 0.001	69 (12.96)	72.5 (15.64)	0.15
Exercise	74.5 (17.38)	135.5 (17.82)	< 0.001	70 (11.76)	63 (12.44)	0.001
Self-monitoring of blood sugar levels						
Foot care	37.25 (8.16)	67.75 (8.91)	< 0.001	34 (7.08)	31.5 (6.22)	0.03
Blood sugar level management during hypoglycemia	37.75 (9.19)	67.75 (8.91)	< 0.001	33.25 (7.29)	31.5 (6.22)	0.21
Management of hyperglycemia	37.25 (8.76))	67.75 (8.91)	< 0.001	33.00 (7.23)	31.5 (6.22)	0.29
Total self-efficacy	372.5 (70.37)	677.5 (89.11)	< 0.001	346.25 (45.94)	328.00 (61.10)	0.07

The results indicate that SMBM significantly enhanced self-efficacy across various domains related to diabetes management when compared to the control group, as evidenced by the p-values (< 0.001). The findings indicate that incorporating SMBM can significantly enhance self-efficacy in individuals managing diabetes, suggesting its potential in improving health outcomes and treatment adherence.

## Discussion

This study investigated the impact of SMBM on the knowledge, self-care behaviors, and self-efficacy of patients with T2D in Zahedan, Iran. The findings demonstrated that SMBM significantly improved self-efficacy across various diabetes management domains compared to the control group. Notably, participants in the SMBM group showed significant improvements in adherence to treatment plans, dietary practices, exercise routines, self-monitoring of blood sugar (SMBG), foot care, and management of both hypoglycemia and hyperglycemia.

These positive outcomes can likely be attributed to the specific design elements of the SMBM intervention. The accessible content and social media platform facilitated knowledge acquisition regarding medication schedules, healthy eating habits, and the importance of adherence [29]. Additionally, peer support and communication with healthcare professionals within the SMBM platform might have enhanced motivation and accountability [30]. For exercise routines, the microlearning approach could have provided clear instructions and guidance suitable for diabetic patients. Furthermore, the social aspects of SMBM might have fostered a sense of community and encouraged participation in physical activity through shared experiences and support [31].

Similarly, SMBM likely equipped participants with the necessary skills and knowledge for self-monitoring of blood sugar, proper foot care practices, and strategies for managing both hypoglycemia and hyperglycemia [30]. The intervention’s real-time communication potential with healthcare professionals could have further addressed individual concerns and ensured proper management techniques. By enhancing knowledge and self-care skills across various domains, SMBM likely contributed to a significant increase in participants’ overall self-efficacy related to diabetes management. Feeling empowered and confident in their ability to manage their condition is crucial for long-term adherence to self-care practices [32].

An interesting observation in this study was the decrease in scores on several self-care parameters within the control group between pre-test and post-test. This decline could be due to several factors. Without the educational intervention and support provided by SMBM, participants in the control group may not have received the necessary information or motivation to maintain or improve their self-care practices over time [13]. Self-care management for chronic conditions like diabetes can be challenging, and it’s possible that participants in the control group experienced a natural decline in adherence or motivation during the study period [6]. Additionally, pre-test scores might reflect temporary improvements in self-care behaviors, and without intervention, participants might regress towards their average self-care practices over time [30]. Future research with longer follow-up periods could explore these possibilities and provide a clearer understanding of how self-care behaviors change over time in the absence of an intervention.

The findings of this study align with previous research on the impact of technology-based educational interventions, specifically those utilizing social media and interactive methods, on the knowledge, self-car of diabetic patients. Through these approaches, participants gained insights into the disease, its complications, and developed crucial skills to effectively manage their condition [31]. Furthermore, the microlearning intervention also resulted in an increase in average self-efficacy in the intervention group compared to the control group. This finding was consistent with studies by Wang et al., and Izquierdo et al. [18, 33]. The effectiveness of Microlearning can be credited to its ability to enhance engagement, accessibility, cater to diverse learning styles, and improve the retention of educational content. The positive influence of SMBM on individuals with T2D stems from its accessible content delivery, fostering community support, facilitating real-time communication with healthcare providers, cost-effectiveness, boosting health literacy, and promoting self-awareness [17]. Although some studies suggest that microlearning may not directly induce behavioral changes, it notably enhances cognitive abilities such as knowledge acquisition and attitude transformation [34]. Future research will focus on refining microlearning elements for optimal behavioral change and improved self-care outcomes. This will involve exploring the optimal duration, frequency, and content of microlearning interventions to maximize their impact on self-care behaviors and overall health outcomes. Additionally, further research will investigate the potential of microlearning to address health disparities and improve access to healthcare for underserved populations.

In addition, the existence of social networks made it possible for the study participants to talk about their experiences and the reasons for adopting self-care behavior. Everyone was also asked to speak of their experiences with self-care behavior. Then the correct way of performing self-care behavior was re-trained for diabetic patients, and diabetic patients independently performed self-care behavior. If each diabetic patient had difficulty performing self-care behavior, additional explanations and training were provided. During the intervention, researchers addressed questions and problems through planned times, encouraging patients to share their ideas and suggestions within the group. This approach fostered a collaborative learning environment, allowing participants to contribute to the discussion and share their experiences, which could have contributed to the overall effectiveness of the microlearning approach in improving self-care and self-efficacy among patients with diabetes [29, 35].

This study had several limitations. Short-term follow-up of the impact of the training program implemented and the collection of information through the questionnaire had limitations؛ This is because some participants may not have provided true information, and another limitation of the present study was that due to the cultural context of the ruling Zahedan city The results of this study do not have the ability to generalize to other places.

## Conclusions

This study investigated the impact of social media-based microlearning (SMBM) on self-efficacy and self-care behaviors in patients with type 2 diabetes (T2D) in Zahedan, Iran. The findings demonstrate that SMBM is an effective intervention for improving self-efficacy across various domains of diabetes management compared to a control group. Participants in the SMBM group showed significant improvements in adherence to treatment plans, dietary practices, exercise routines, self-monitoring of blood sugar (SMBG), foot care, and management of both hypoglycemia and hyperglycemia. Future research can explore how to refine SMBM elements for optimal long-term behavioral change and investigate its effectiveness in promoting self-care in diverse populations.

## Data Availability

The data that support the findings of this study are available from the corresponding author on request.
